# HPV-mediated down-regulation of NOD1 inhibits apoptosis in cervical cancer

**DOI:** 10.1186/s13027-020-0272-3

**Published:** 2020-01-30

**Authors:** Xubin Liu, Hanyu Ma, Lingyan Fei, Mengjie Jiang, Meng Xia, Lihong Bai, Xufang Pi, Shangwu Chen, Li Yu

**Affiliations:** Department of Pathology, The First Affiliated Hospital, Sun Yat-sen University, Guangzhou, 510080 People’s Republic of China

**Keywords:** NOD1, Cervical intraepithelial neoplasia, Cervical cancer, HPV16 E6/E7, Apoptosis

## Abstract

Cervical cancer is the fourth most common malignant tumor in women worldwide. The persistent infection of high-risk Human Papillomavirus (hrHPV) is considered to be the primary cause of this disease. As an innate immune receptor, the nucleotide-binding oligomerization domain protein-1 (NOD1) recognizes the pathogen-associated molecular pattern (PAMP), subsequently initiating immune responses. NOD1 is also involved in the apoptotic signaling pathway and mutates in many cancer cells. In the study, we revealed that NOD1 expression decreased during the progression of cervical intraepithelial neoplasia to cervical cancer and that HPV16 E6/E7 oncoproteins induced down-regulation of NOD1. Moreover, the activation of NOD1 promoted the apoptosis of HPV16-positive cervical cancer cells. The data indicated that the dysregulation of NOD1-mediated inflammation and apoptosis may contribute to cervical intraepithelial neoplasia progression and cervical cancer.

## Introduction

Cervical cancer is the fourth most common cancer in women, with a high mortality rate [1]. Although cervical cancer can be prevented by screening tests and Human Papillomavirus (HPV) vaccines in most western countries, it is still the third most common cancer in developing countries [2]. High-risk human papillomavirus (hrHPV) persistent infection is considered the main risk factor for cervical cancer development [3]. E6 and E7 are the major oncoproteins of hrHPV. E6 can direct p53 ubiquitin-mediated degradation, while E7 binds to Rb protein and regulates the G1/S checkpoint. E6 co-operation with E7 promotes the development of cervical cancer [4]. About 90% of HPV infections are eliminated within 3 years [5]. Only a few patients may develop cervical squamous intraepithelial lesion (SIL), and less than 1% of patients will progress to cervical cancer [6]. Therefore, in addition to HPV infection, other factors such as smoking and oral contraceptives may also be related to the occurrence of cervical cancer [7].

Inflammation is another important risk factor in cervical cancer development [8, 9]. The pattern-recognition receptors (PRRs) recognize pathogen-associated molecular patterns (PAMPs), triggering the intracellular signaling cascades and inducing the transcriptional expression of inflammatory mediators that can eliminate the invading pathogens [10, 11]. The nucleotide-binding oligomerization domain protein-1 (NOD1) is one of the most important members of the NOD-like receptor (NLR) family, a type of PRR [12]. Like other NLRs, NOD1 consists of multiple leucine-rich repeats (LRRs), a nucleotide-binding oligomerization domain, and a caspase activation and recruitment domain (CARD) [13]. NOD1 is a cytosolic protein that recognizes the PAMPs on bacterial cell walls, such as peptidoglycan (PGN) and lipopolysaccharide (LPS) [10, 14]. The recognition of PAMPs by NOD1 leads to the activation of the NF-κB pathway, which drives the proinflammatory and antimicrobial responses [14, 15]. Since epithelial cells are the first barrier against pathogens, NOD1 has functional expression in epithelial cells of human digestive and reproductive systems [16]. Several studies suggested that NOD1 plays crucial role in the development of cancers, such as gastric cancer colorectal cancer, and breast cancer [17, 18]. NOD1 was found involved in TNF-induced apoptosis and the overexpression of NOD1 could enhance sensitivity to TriDAP-induced apoptosis in breast cancer cells [18]. Moreover, NOD1 shows tumor suppressor properties in colon [19]. NOD1 could protect the intestinal epithelial cells from injury, bacterial translocation, and colitis by regulating cell survival [19]. NOD1 dysfunction resulted in inflammation, increasing the risk of colitis-associated colon cancer [19]. As the cervix is often in contact with pathogenic microorganisms, we speculate that NOD1-related signal pathway may be involved in the occurrence of cervical cancer. In the current study, we investigated the expression of NOD1 in different grade cervical lesions, the impact of HPV infection on NOD1 expression, and the role of NOD1-mediated signaling in cervical cancer.

## Materials and methods

### Patients and tissue sampling

A total of 243 formalin-fixed, paraffin-embedded tissue samples were used in this study, including 50 cervical intraepithelial neoplasia (CIN) I, 52 CIN II, 44 CIN III, 61 invasive squamous cell carcinomas (ISCCs) samples and 36 normal control cervical tissues obtained from surgically removed uteruses that were reported to be either hysteromyoma or adenomyosis. The tissues were sectioned and histologically diagnosed by pathologists at the Department of Pathology, the First Affiliated Hospital of Sun Yat-Sen University. ISCCs were diagnosed when the epithelial basement membrane was breached. In addition, 30 frozen samples including 20 normal cervical epithelia and 10 ISCCs tissues were included in the study. None of the patients has received radiotherapy or chemotherapy.

### Immunohistochemistry

Sections (4 μm) were obtained from formalin-fixed, paraffin-embedded normal (*n* = 36), CIN I (*n* = 50), CIN II (*n* = 52), CIN III (*n* = 44), and ISCCs (*n* = 61) tissues. Immunohistochemistry (IHC) staining was conducted as described previously [20] with antibodies against NOD1 (MAB7090, monoclonal mouse IgG2B clone, diluted at 1/60; R&D Systems, Minneapolis, USA), p16^INK4A^ (p16^INK4A^ (F-12): sc-1661, mouse monoclonal antibody, diluted at 1/100, Santa Cruz Biotechnology Inc., California, USA), and horseradish peroxidase-labelled secondary antibody (Maixin Biotechnology, Fuzhou, China) in accordance with manufacturer’s instructions. Color was developed with diaminobenzidine (Dako Corp, CA 95051, USA) incubated for 5–10 min at room temperature. The double immunostaining of NOD1 and p16^INK4A^ was performed with DouSPTM double staining kit (MaxVision, Fuzhou, China). Slides were counterstained with haematoxylin and photos were taken by light microscopy.

Staining intensity was graded according to the following criteria described in previous studies [20, 21]: (−), no positive staining cells; (1+), less than 25% positive staining cells with weak intensity and focal distribution; (2+), 26–50% positive staining cells with moderate intensity and focal distribution; and (3+), more than 50% positive staining cells with strong intensity and diffuse distribution.

### Cell culture

The cervical squamous cell cancer-derived cell lines C33A, SiHa, and ME180 were purchased from Shanghai Institute for Biological Sciences (Shanghai, China). SiHa and C33A cells were cultured in Minimum Essential Medium (MEM) (GIBCO, Shanghai, China) and ME-180 cells were cultured in Dulbecco’s Modified Eagle Medium (DMEM) (GIBCO, Shanghai, China) in a humidified atmosphere containing 5% CO_2_ at 37 °C, supplemented with 10% fetal bovine serum (GIBCO).

### Cell transfection

The HPV-negative C33A cells were seeded in 6-well plates for 24 h before transfection. Cells grown to 50–70% confluency were transfected using Lipofectamine®2000 (Invitrogen, Shanghai, China) for HPV16 wild type E6 and E7 gene transfection. Cells were divided into four groups for treatment: the blank group (without plasmid), the negative control (empty vector) group, the E6 group (E6-expressing plasmid), and E7 group (E7-expressing plasmid). A volume of 20 μl Lipofectamine®2000 reagent was used per microgram of DNA. Plasmids were constructed with the pEGFP-N1 vector. All of these plasmids were kindly provided by Prof. Xudong Tang (Institute of Biochemistry and Molecular Biology, Guangdong Medical University).

### Immunocytochemistry

Cells were grown on sterile glass coverslips or 35 mm culture dishes. The adherent cells were rinsed briefly in phosphate-buffered saline (PBS) and incubated in 4% paraformaldehyde in PBS (pH 7.4) for 15 min at room temperature. After washing 3 times with PBS, the cells were incubated with PBS containing 0.5% Triton X-100 for 20 min, then blocked in 1% BSA in PBST (PBS with 0.1% Tween 20) for 30 min, and incubated in the diluted antibodies against NOD1 and p16^INK4A^ overnight at 4 °C. Color was developed as described in immunohistochemistry section.

### Cell apoptosis assay

Cell apoptosis was evaluated by flow cytometry with Annexin V-FITC assay kit (KeyGEN BioTECH, Nanjing, China). Approximate 3 × 10^5^ typsin pre-treated cells were centrifuged at 2000 rpm for 5 min, rinsed twice with ice-cold PBS, and resuspended in Annexin binding buffer containing Annexin V-FITC and propidium iodide. Cells were incubated at room temperature for 15 min and analyzed using a Becton Dickinson FACScan (BD Biosciences, San Jose, California, USA). Cycloheximide (CHX) was obtained from Sigma (St. Louis, Missouri, USA). γ-D-Glu-mDAP (iE-DAP) was purchased from Invivogen (Carlsbad, California, USA).

### Real-time quantitative reverse transcription PCR

Total RNA was extracted from cultured cells or fresh cervical tissues using Trizol reagent (Invitrogen, Shanghai, China) according to the manufacturer’s protocol. Total RNA (1 μg) was converted to cDNA with Transcriptor First Strand cDNA Synthesis Kit (Roche, Mannheim, Germany). The primers for NOD1, RIP2, and GAPDH were synthesized by Sangon (Shanghai, China). The primer sequences were 5′-CCACTTCACAGCTGGAGACA-3′ (forward) and 5′- TGAGTGGAAGCAGCATTTTG-3′ (reverse) for NOD1; 5′-ACGTCTGCAGCCTGGTATAGC-3′ (forward) and 5′-CATCTAGCGACTGGTTAAG-3′ (reverse) for RIP2; and 5′-AGAAGGCTGGGGCTCATTTG-3′ (forward) and 5′- AGGGGCCATCCACAGTCTTC-3′ (reverse) for GAPDH. qPCR was carried out in triplicate in a 20 μl reaction volume using FastStart Universal Probe Master (ROX) (Roche, Mannheim, Germany) and Applied Biosystems 7500 Real-Time PCR System (Applied Biosystems, Foster City, California, USA). The relative quantity of the target mRNA was normalized to the level of GAPDH mRNA level. Relative gene expression was evaluated using the 2^-ΔΔCt^ method [22].

### Western blot

The proteins of cells or fresh tissues were extracted using Whole Cell Lysis Assay (KeyGEN BioTECH, Nanjing, China). Proteins concentration was determined using BCA Protein Assay Kit (Beijing CoWin Biotech, Beijing, China). Proteins (30μg) were separated on 10% sodium dodecyl sulfate-polyacrylamide gel electrophoresis (SDS-PAGE) gel and then transferred to polyvinylidene difluoride (PVDF) membranes (Millipore, Burlington, Massachusetts, USA). Membranes were blocked with 5% nonfat dried milk and incubated overnight with antibodies against NOD1 (MAB7090, 1:500; R&D Systems, Minneapolis, Minnesota, USA) or GAPDH (#2118, 1:8000; Cell Signaling Technology, Danvers, Massachusetts, USA). Membranes were then incubated with horseradish peroxidase-conjugated secondary antibodies for 60 min. Immunoreactive bands were detected by using Immobilon Western Chemiluminescent HRP Substrate (Millipore, Burlington, Massachusetts, USA).

### Statistical analysis

IBM SPSS 22.0 and GraphPad Prism 6.01 were used for statistical analysis of the data. The Kruskal–Wallis test was used to compare the differences of cumulative NOD1 and p16^INK4A^ expression with CIN progression and ISCCs among the sample groups. The multiple comparisons between every two groups were examined by Bonferroni test, and a *p*-value ≤0.05 was considered statistically significant. The correlation of NOD1 and p16^INK4A^ expression was examined by Spearman correlation test. Student’s *t*-test was used to compare the differences of NOD1 mRNA expression in normal cervical epithelia, CINs, and ISCCs.

## Result

### NOD1 expression decreased with CIN progression

**Fig. 1:**
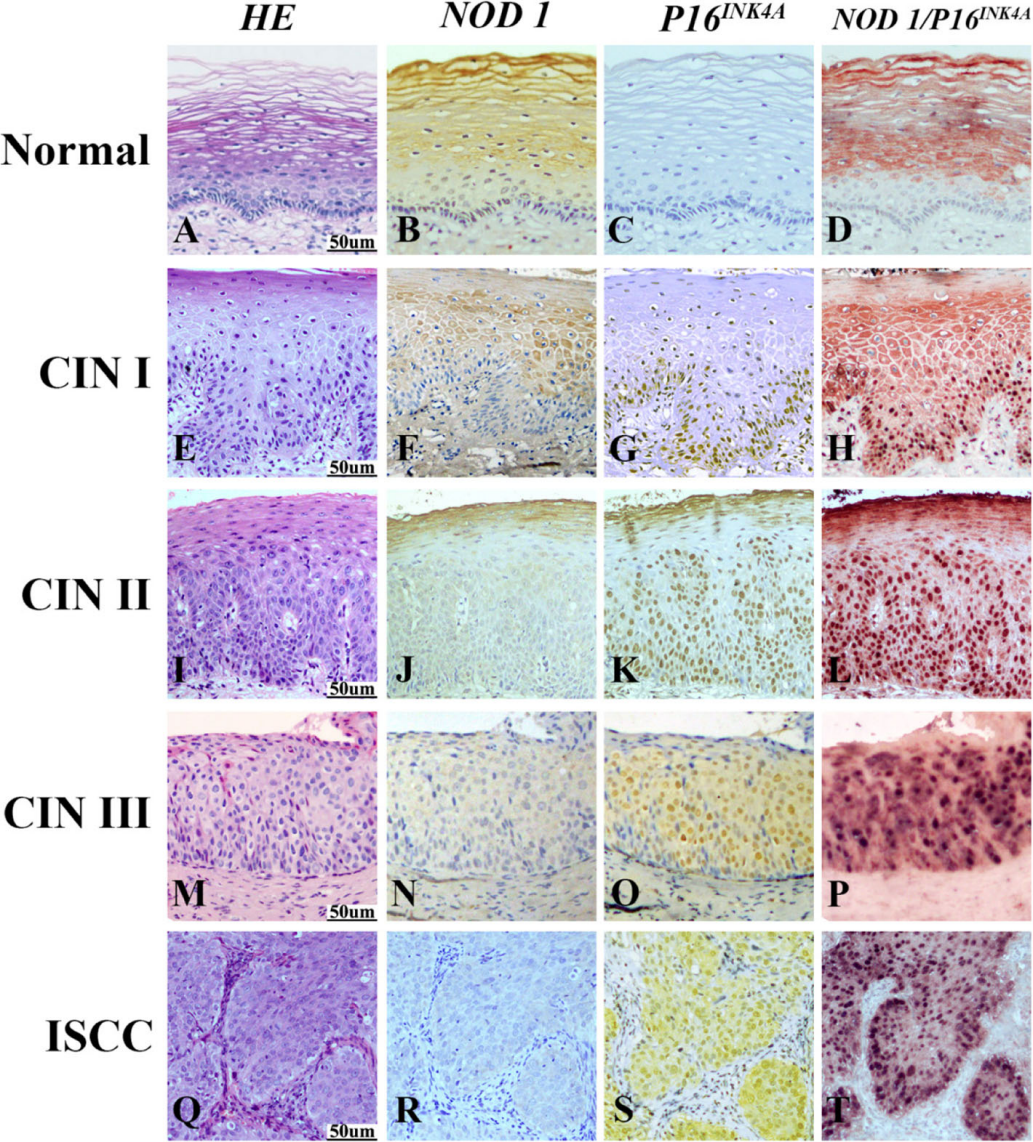
Expression of NOD1 and P16^INK4A^ in normal cervix, CIN I, CIN II, CIN III and ISCC tissues detected by immunohistochemistry. Hematoxylin and eosin (HE) staining showed that in comparison to normal cervical squamous epithelium (**a**), atypical hyperplasia of squamous epithelium was restricted within lower third in CIN I (**e**), lower two-thirds in CIN II (**i**), or exceeded over lower two-thirds in CIN III (**m**), respectively. Carcinoma invaded into muscle tissue in ISCC (**q**). NOD1 showed the strongest immunoreactivity in normal cervical squamous epithelium (**b**), and the staining intensity of NOD1 gradually decreased from CIN I (**f**), CIN II (**j**) to CIN III (**n**). No NOD1 immunostaining was observed in cells within carcinoma nests (**r**). In contrast to NOD1, no p16^INK4A^ staining was detected in the normal cervical tissue (**c**). The expression of p16^INK4A^ gradually increased from CIN I (**g**), CIN II (K) to CIN III (**o**), and p16^INK4A^ positive cells were diffusely distributed within the atypical hyperplasia tissue. Diffuse and strong staining of p16^INK4A^ was observed in tumor epithelial cells of carcinoma nests in ISCC (S). NOD1 staining was only detected in normal cervical squamous epithelium (**d**) with double staining of NOD1 (red) and p16^INK4A^ (black), and NOD1 staining was not observed within atypical hyperplasia cells of CINs (H, L, P), where p16^INK4A^ was positive. Strong p16^INK4A^ staining but no NOD1 staining was observed in the tumor cells of carcinoma nests (**t**)

**Fig. 2:**
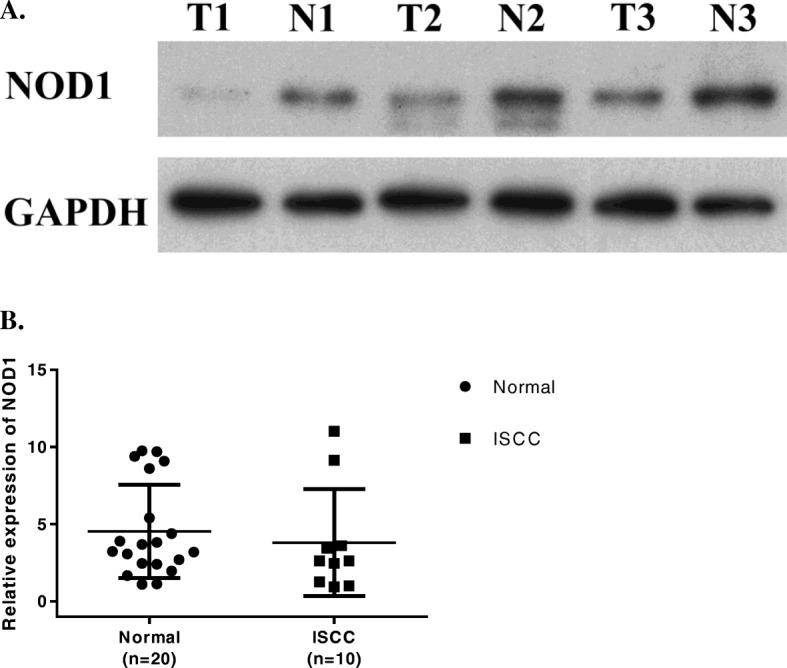
NOD1 expression in frozen fresh ISCC and normal cervical tissues. **a.** Expression of NOD1 protein in 3 paired tumor (T) and adjacent normal (N) tissues from 3 patients was detected by Western blot. **b.** Quantitative comparison of the NOD1 mRNA expression between ISCC and normal cervical tissues using RT-PCR with GAPDH as the control house keeping gene. The ratios of NOD1/GAPDH expression were calculated from triplicate. (*P* = 0.22)

In order to understand the possible function of NOD1 in cervical cancer, we first profiled its expression in CINs and ISCCs tissues by immunostaining. We found that 97.2% of the normal cervix (35/36) and 86.0% of CIN I samples (43/50) were stained positive for NOD1 (Table 1). The 47.9% of high-grade squamous intraepithelial lesion (HSIL) (46/96) consisting of CIN II and CIN III and 14.8% of ISCCs (9/61) were positive for NOD1. In term of immunostaining intensity, the strong NOD1 expression (2+ and 3+) was detected in 77.8% normal cervical, 30.0% CIN I, 9.6% CIN II, and 6.8% CIN III samples, respectively, while no strong staining was observed in ISCCs (Fig. 1, Table 1). Our results indicated that NOD1 positive rates, particularly immunostaining intensity, gradually decreased along with the progression of cervical lesions. When NOD1 protein was examined using Western blot in 3 paired tumor (T) and adjacent normal (N) tissues from cervical cancer patients, NOD1 level in normal tissues was higher than that in cancer tissues (Fig. 2a). Compared with normal cervical tissues, the expression of NOD1 mRNA in cervical cancers decreased (Fig. 2b), although the difference was not statistically significant.

**Table 1:** Expression of NOD1 in CINs and ISCCs

Tissues	Degree of immunoreactivity(%)			
	–	1+	2+	3+
Normal^a^	1/36 (2.8)	7/36 (19.4)	15/36 (41.7)	13/36 (36.1)
CIN I^b^	7/50 (14.0)	28/50 (56.0)	14/50 (28.0)	1/50 (2.0)
CIN II^c^	25/52 (48.1)	22/52 (42.3)	5/52 (9.6)	0/52 (0.0)
CIN III^d^	25/44 (56.8)	16/44 (36.4)	3/44 (6.8)	0/44 (0.0)
ISCCs^e^	52/61 (85.2)	9/61 (14.8)	0/61 (0.0)	0/61 (0.0)

*CIN* cervical intraepithelial neoplasia, *ISCCs* invasive squamous cell carcinomas

^a-b^*P* < 0.005, ^a-c^*P* < 0.005, ^a-d^*P* < 0.005, ^a-e^*P* < 0.005, ^b-c^*P* < 0.005, ^b-d^*P* < 0.005,

^b-e^*P* < 0.005, ^c-d^*P* > 0.05, ^c-e^*P* < 0.005, ^d-e^*P* < 0.05;

### NOD1 expression was inversely correlated to expression of p16^INK4A^

We investigated whether NOD1 expression is related to p16INK4A expression. We found that more than 98% CINs and ISCCs samples were stained positive for p16^INK4A^, but none of the normal cervical tissues was stained positive (Table 2). An increased intensity of p16^INK4A^ staining was also observed along with the increased histopathologic grade of cervical intraepithelial lesions (Fig. 1). The differences of positive staining levels for p16^INK4A^ among normal controls, CINs, and ISCCs were statistically significant, and so was CINs and ISCCs (*P* < 0.005) (Table 2). When double immunostaining of NOD1 and p16^INK4A^ was performed, NOD1 showed high expression in normal cervical tissues and gradually decreased expression with the increased histopathologic grade in CINs, while p16^INK4A^ expression increased with the progression from CINs to ISCCs. Interestingly, no NOD1 expression was detectable in p16^INK4A^ positive cells (Fig. 1). The results indicated that the expression of NOD1 was inversely correlated to the expression of p16^INK4A^ (rs = − 0.382**,** *P* < 0.001) (Table 3).

**Table 2:** Expression of p16^INK4A^ in CINs and ISCCs

Tissues	Degree of immunoreactivity(%)			
	–	1+	2+	3+
Normal^a^	36/36 (100.0)	0/36 (0.0)	0/36 (0.0)	0/36 (0.0)
CIN I^b^	1/50 (2.0)	31/50 (62.0)	15/50 (30.0)	3/50 (6.0)
CIN II^c^	1/52 (1.9)	5/52 (9.6)	27/52 (51.9)	19/52 (36.5)
CIN III^d^	0/44 (0)	6/44 (13.6)	25/44 (56.8)	13/44 (29.5)
ISCCs^e^	0/61 (0)	16/61 (26.2)	14/61 (23.0)	31/61 (50.8)

*CIN* cervical intraepithelial neoplasia, *ISCCs* invasive squamous cell carcinomas

^a-b^*P* < 0.005, ^a-c^*P* < 0.005, ^a-d^*P* < 0.005, ^a-e^*P* < 0.005, ^b-c^*P* < 0.005, ^b-d^*P* < 0.005,

^b-e^*P* < 0.005, ^c-d^*P* > 0.05, ^c-e^*P* > 0.05, ^d-e^*P* > 0.05;

**Table 3:** A cross tabulation of NOD1 and p16^INK4A^ expression

		NOD1 expression level (cases)				Total
		–	1+	2+	3+	
p16^INK4A^ expression level (cases)	–	5	7	14	12	38
	1+	23	20	13	2	58
	2+	46	31	4	0	81
	3+	36	24	6	0	66
Total		110	82	37	14	243

243 specimens that include 146 CINs, 61 ISCCs and 36 controls

Correlation of NOD1 and P16^INK4A^ expression: rs = −0.382, *p* < 0.001

### HPV16 E6/E7 down-regulated the expression of NOD1

**Fig. 3:**
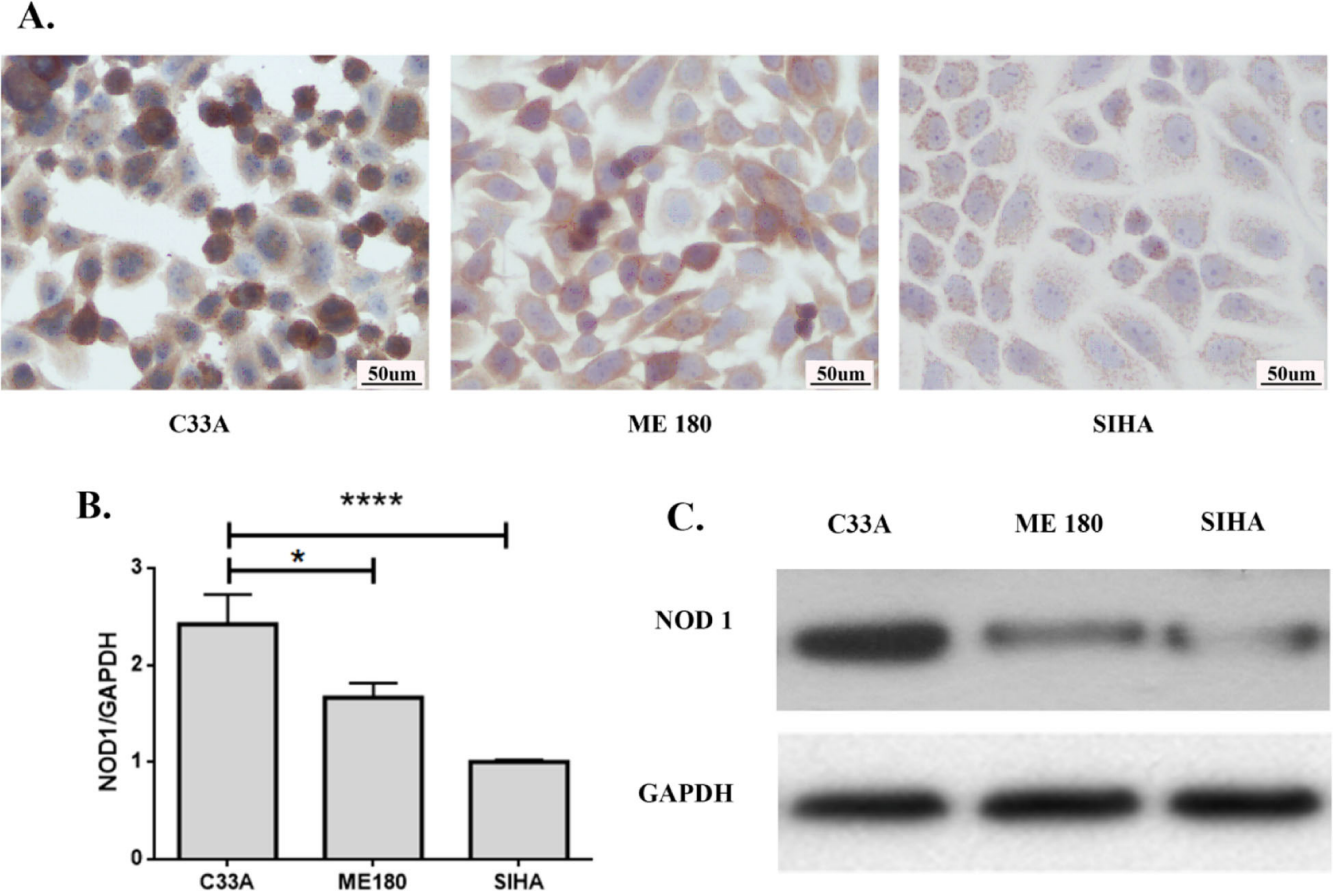
Expression of NOD1 in 3 cervical squamous carcinoma cells was detected by immunocytochemistry (**a**), quantitative real-time PCR (**b**) and Western blot (**c**). NOD1 showed strongest expression in the HPV (−) C33A cells and was weakest in the HPV16 (+) SiHa cells. *P < 0.05, ****P < 0.0001

Since the expression of NOD1 decreased with the progression of CINs, and was negatively correlated to the expression of p16^INK4A^, we analyzed whether the expression of NOD1 was related to hrHPV infection. The expression of NOD1 was examined by immunocytochemistry in 3 cervical cancer cells: SiHa infected with type 16 HPV, the predominant HPV type (46–63%) in cervical squamous cell carcinoma [23]; ME180 infected with type 68 HPV; and C33A without HPV infection. The NOD1 immunostaining intensity was stronger in HPV-negative C33A cells compared to HPV16-positive SiHa cells and HPV68-positive ME180 cells (Fig. 3a). Immunocytochemistry results were confirmed by RT-PCR and Western blot (Fig. 3 b, c), suggesting that HPV, especially hrHPV (type16), may be related to the down-regulation of NOD1 in cervical cancer cells.

**Fig. 4:**
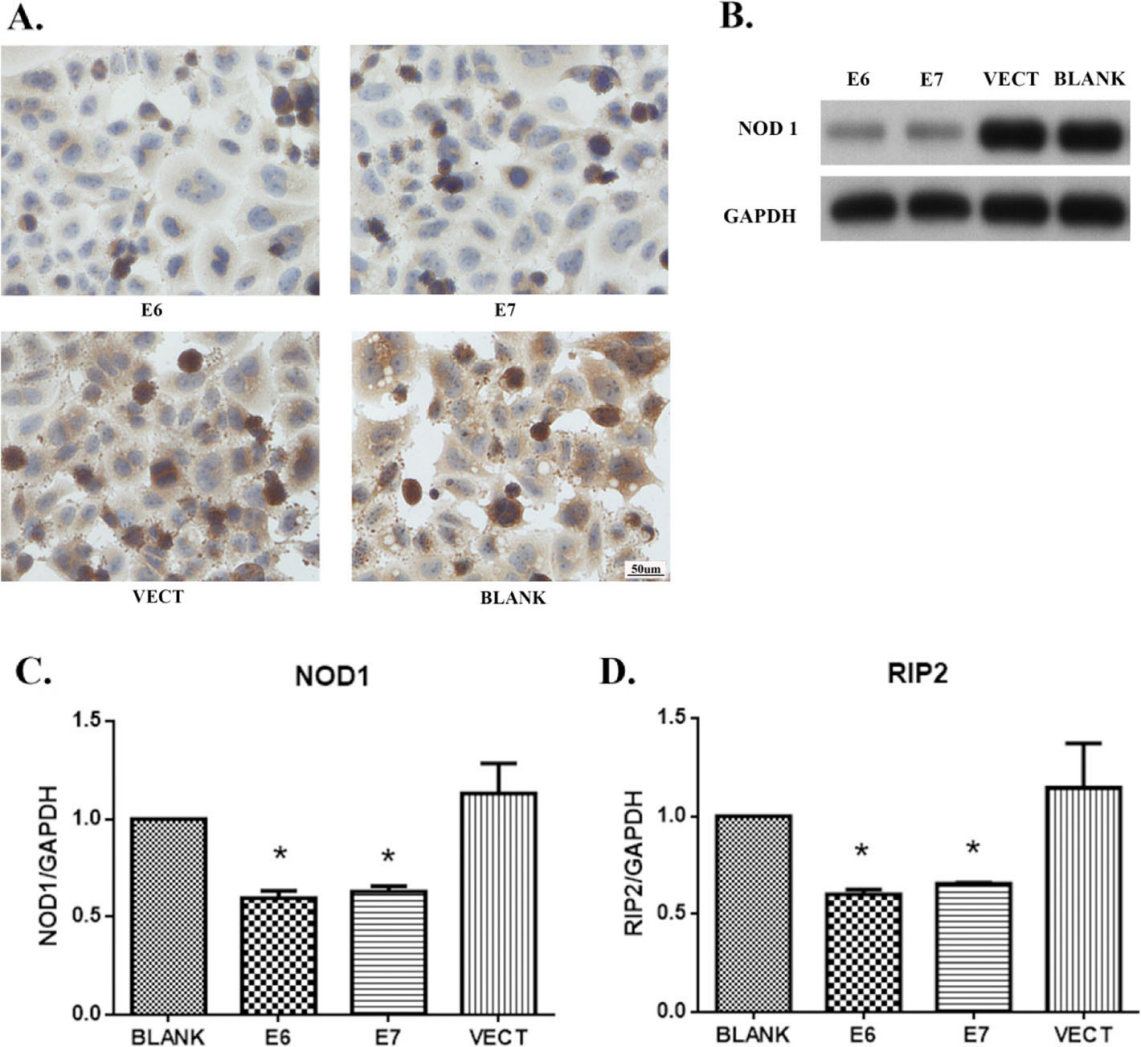
HPV oncoproteins down-regulated the expression of NOD1 and RIP2 in C33A cells. NOD1 expression was down-regulated in the cancer cells transfected with E6- or E7-expressing plasmids detected by immunocytochemistry (**a**), Western blot (**b**), and RT-PCR (**c**). RIP2 mRNA expression was down-regulated in the cancer cells transfected with E6- or E7-expressing plasmids detected by RT-PCR (**d**). BLANK, without plasmid, VECT, empty vector, E6, E6-expressing plasmids, and E7, E7-expressing plasmids

To explore the mechanism of down-regulation of NOD1 expression, HPV-negative C33A cells were transfected with E6 and E7 expressing plasmids or the empty vectors (VECT). When HPV16 E6 or E7 was ectopically expressed in C33A cells, the NOD1 expression decreased significantly in both protein (Fig. 4a, b) and mRNA levels (Fig. 4c). RIP2 mRNA level was also significantly down-regulated in HPV16 E6/E7-expressing cells (Fig. 4d**)**.

### The activation of NOD1 increased CHX-induced apoptosis

**Fig. 5:**
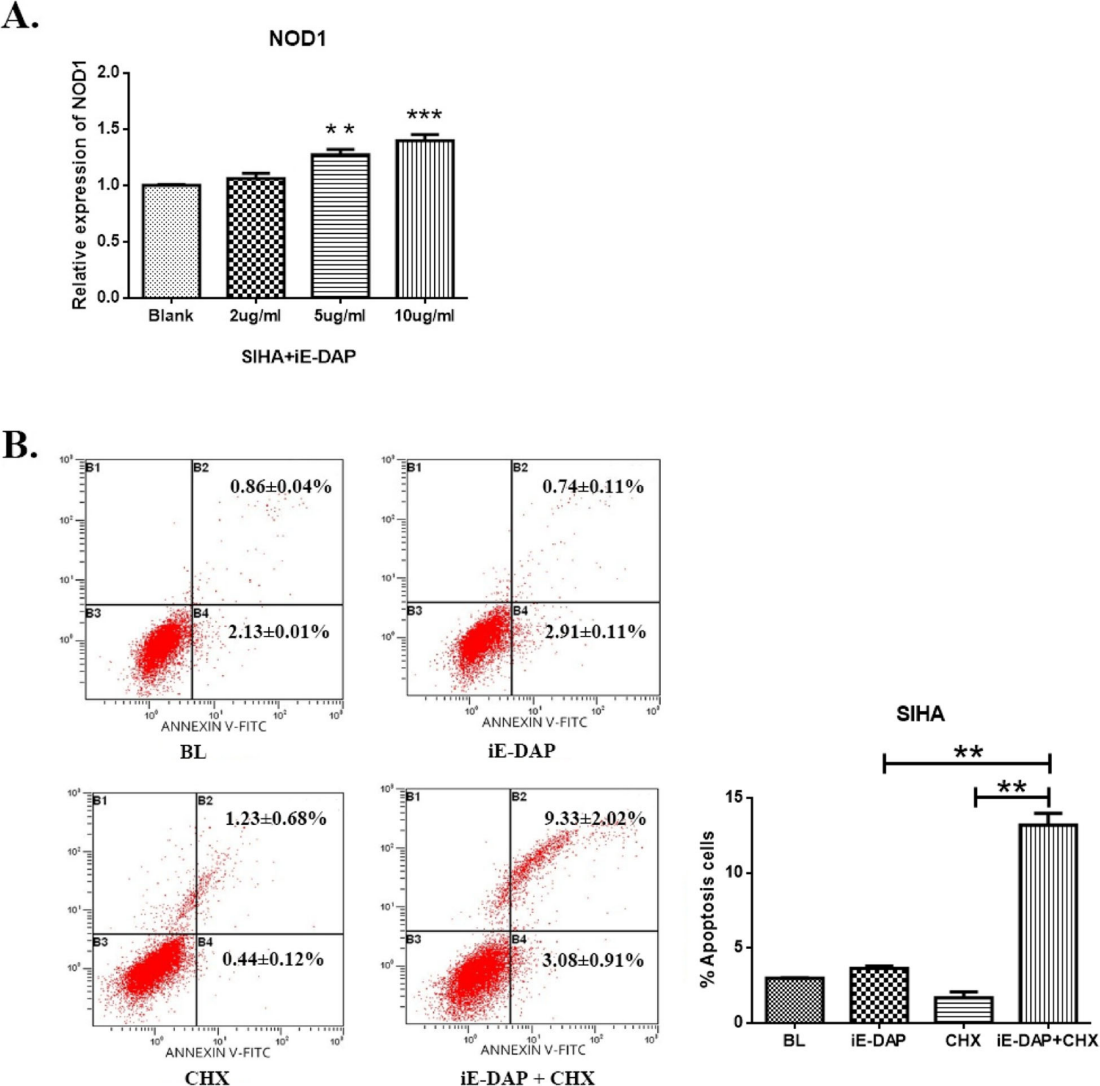
The activation of NOD1 increased CHX-induced apoptosis. NOD1 expression increased in SiHa cells treated with iE-DAP (**P < 0.005, ***P < 0.0001) (**a**). The NOD1 activation enhanced CHX-induced apoptosis. HPV16-positive SiHa cells were treated with iE-DAP in the presence or absence of CHX. Cell viability was measured by flow cytometry. The apoptosis of SiHa cells increased significantly in the presence of CHX (*P* < 0.005) (**b**)

The reduction of apoptosis and dysregulation of cell proliferation caused by hrHPV are the main cause of cervical cancer. We investigated the effect of NOD1 on apoptosis of hrHPV-infected cervical cancer cells. When HPV16-positive SiHa cells were stimulated with iE-DAP, the mRNA level of NOD1 was positively correlated with the dose of iE-DAP (Fig. 5a). In the presence of CHX, the exposure of SiHa cells to iE-DAP induced about 13% cell death, which was significantly higher than that of iE-DAP or CHX alone (*P* < 0.005) (Fig. 5b). The results indicated that NOD1 can enhance the sensitivity of HPV16-positive cells to apoptosis induced by CHX and the decrease of NOD1 expression may contribute to the apoptosis resistance and the development of cervical cancer.

## Discussion

Human papillomavirus infection is an essential causal factor of cervical cancer, but the role of other etiological factors is unclear [24]. Cervix is often exposed to bacteria, and Gram-negative bacteria-related inflammation has been reported [20]. NOD1, an innate immune receptor, recognizes specific molecular patterns expressed on the cell walls of Gram-negative bacteria and initiates inflammatory response [12]. Our results demonstrated that HPV infection induced down-regulation of NOD1 in cervical intraepithelial lesions and ISCCs.

hrHPV E6 and E7 oncoproteins have been found to inhibit the expression of Toll-like receptor 9 (TLR9), an innate immune receptor of TLRs family [25]. p16^INK4A^ is a surrogate marker for the infection of hrHPV, and p16^INK4A^ immunostaining has been suggested as an alternative approach to histology review, which is valuable for confirming the cervical intraepithelial lesions in clinicopathologic diagnosis [26–28]. We previously found that the expression of TLR4 was inversely correlated to p16^INK4A^ in cervical intraepithelial lesions and ISCCs [20]. The HPV16 oncoproteins-mediated suppression of NOD1 may attenuate the pathogen-induced inflammation and subsequent elimination of pathogens.

In addition to innate immune response, NOD1 is also involved in the activation of the apoptosis signaling pathway through its interaction with the CARD domain of RIP2. RIP2 is considered to be an important component of the multiprotein signaling complex that mediates innate immune responses induced by the activation of NOD1 [29, 30]. The abnormal expression of NOD1 and RIP2 has been associated with cell apoptosis and development of breast cancer and oral cancer [18, 31]. The activation of NOD1 by ligand up-regulates the RIP2 expression and turns on the downstream signaling pathways, which in turn initiates multiple cellular responses including the TAK1-mediated release of proinflammatory cytokines and NOD1-dependent apoptosis through caspase 8 [32]. iE-DAP is a dipeptide in bacterial peptidoglycan (PGN), and recognition of iE-DAP by NOD1 can induce a signaling cascade that promotes the activation of NF-κB and the production of inflammatory cytokines [29]. We found that NOD1 in cervical cancer cells can be activated by iE-DAP, consequently facilitating the CHX-induced apoptosis. Cycloheximide is a glutarimide antibiotic that blocks the translation of mRNA and promotes apoptosis through inhibiting the expression of antiapoptotic molecules such as FLIP [33, 34]. Our results suggested that the down-regulation of NOD1 by HPV16 E6/E7 can reduce cell apoptosis, which may promote the development and progression of cervical cancer.

Previous studies have shown that NOD1 signaling protects the gut against injury and inflammation-induced tumorigenesis by maintaining the integrity of the intestinal epithelium. Similar to the gut, the cervix is exposed to a bacterial environment, including symbionts and pathogens that can cause cervicitis. It has been confirmed that the chronic inflammation of the cervix may be related to high-grade CIN development. Combined with the down-regulation of NOD1 expression in CINs and ISCCs, we suggest that the dysregulation of NOD1-mediated inflammation may be an important risk factor in cervical intraepithelial neoplasia and cervical cancer.

## Data Availability

The raw/processed data required to reproduce these findings cannot be shared at this time as the data also forms part of an ongoing study.
